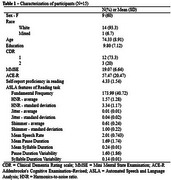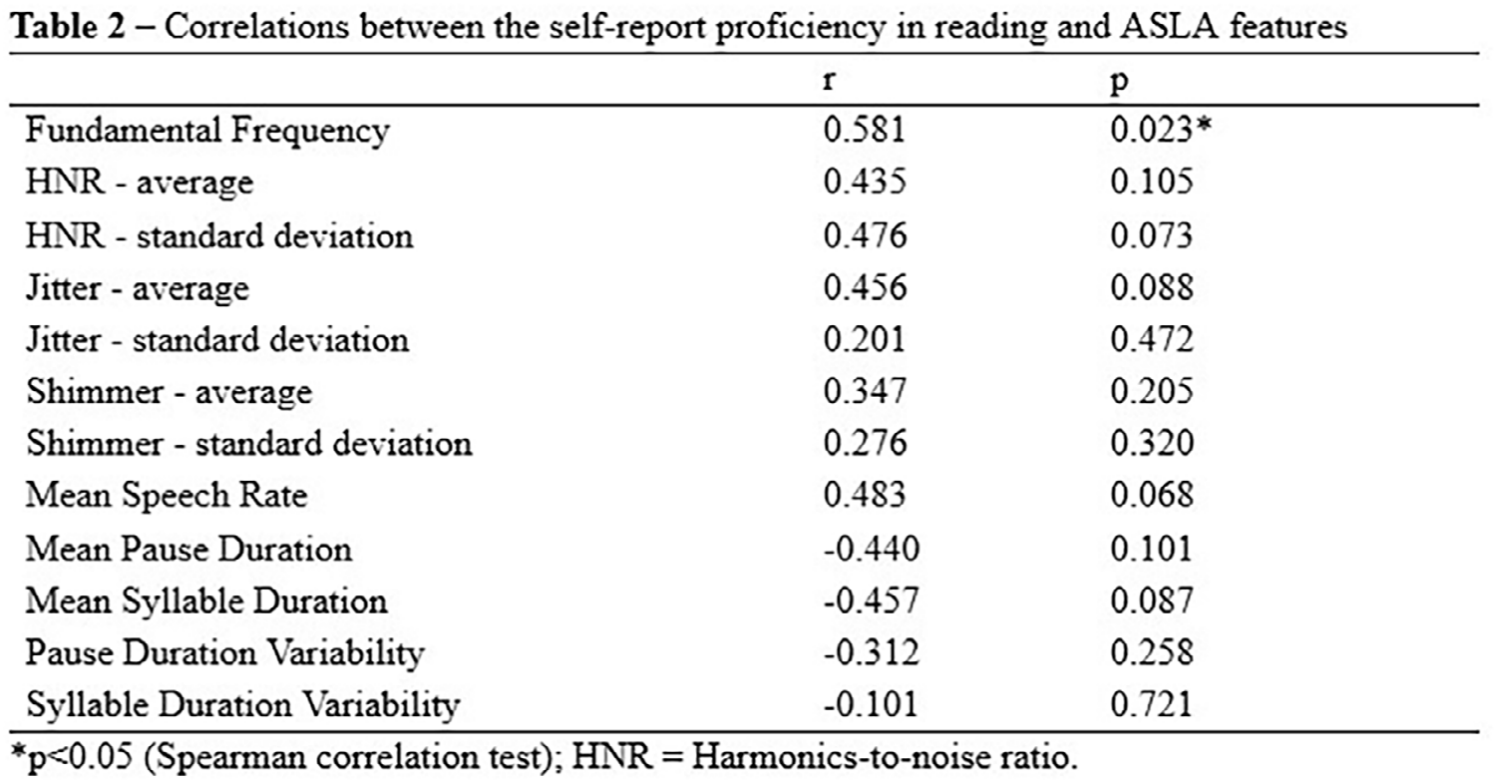# Digital and self‐reported reading measures in Brazilians with Alzheimer's disease: An exploratory correlational study

**DOI:** 10.1002/alz70857_100416

**Published:** 2025-12-24

**Authors:** Guilherme Briczinski Souza, Daniel Coswig Zitzke, Gabriela Sanches, André Luiz Rodrigues Palmeira, Liana Lisboa Fernandez, Adolfo M. Garcia, Bárbara Costa Beber

**Affiliations:** ^1^ Universidade Federal de Ciências da Saúde de Porto Alegre (UFCSPA), Porto Alegre, Rio Grande do Sul, Brazil; ^2^ Universidad Santiago de Chile, Estación Central, Santiago de Chile, Chile; ^3^ Global Brain Health Institute, University of California, San Francisco, CA, USA; ^4^ Cognitive Neuroscience Centre, University of San Andres, Victoria, Buenos Aires, Argentina

## Abstract

**Background:**

Alzheimer's disease (AD) leads to cognitive decline and also affects reading and writing abilities, which are crucial for daily functioning. When a patient presents with reading complaints, it is important to identify the nature of the complaint in order to make an accurate diagnosis and develop a rehabilitation plan. Simple, quick, and accurate assessments are increasingly a necessity in clinical and research settings. In this regard, digital speech markers are emerging as a promising tool for assessing speech and voice tasks, including reading. This study aims to explore correlations between digital speech markers in a reading task and self‐reported reading outcomes in a sample of Portuguese‐speaking Brazilians with AD.

**Method:**

Data were collected between March and December 2024 from literate individuals diagnosed with AD according to NINCDS‐ADRDA criteria. Participants completed a self‐report questionnaire adapted from the Bilingual Language Profile, scoring speaking proficiency and perceived difficulties on a Likert scale (0–6, higher values indicate better proficiency). Speech samples were collected via a paragraph reading task on the Toolkit to Examine Lifelike Language (TELL), a validated speech testing app, using standardized recording conditions. We targeted speech‐timing, pitch‐related, and voice‐quality features (Table 1). Associations between these variables and self‐report scores were tested using Spearman's correlations, with a significance level of 0.05.

**Result:**

The sample comprised 15 AD individuals, with a mean self‐reported reading proficiency of 4.33 (±1.54) –Table 1. A significant correlation was found between self‐reported reading proficiency and fundamental frequency (*r* = ‐0.581, *p* = 0.023) (Table 2).

**Conclusion:**

We found that the lower the reading proficiency, the higher the fundamental frequency. This suggests a possible relationship between self‐perception of reading ability and motor aspects of speech, more specifically voice. These are preliminary results from an exploratory study to better understand the value of digital speech markers. Future analyses with a larger sample and more sophisticated analysis will be conducted, controlling for potential confounders such as age, education level, and disease severity.